# Macrophage plasticity and glucose metabolism: the role of immunometabolism in pulmonary arterial hypertension

**DOI:** 10.1042/CS20257363

**Published:** 2025-10-01

**Authors:** Lindsay Jefferson, Patricia D.A Lima, Stephen L. Archer

**Affiliations:** 1 Department of Medicine, Queen’s University, Kingston, ON, K7L 3N6, Canada; 2 Queen’s Cardiopulmonary Unit, Queen’s University, Kingston, ON, K7L 3N6, Canada; 3 Translational Institute of Medicine, Kingston, ON, K7L 3N6, Canada

**Keywords:** interleukin 1 beta (IL-1β), itaconate, mitochondrial metabolism, NOD-like receptor protein 3 (NLRP3), pyruvate dehydrogenase kinase, succinate dehydrogenase

## Abstract

Pulmonary arterial hypertension (PAH) is a syndrome characterized by a mean pulmonary artery pressure >20 mmHg and elevated pulmonary vascular resistance >2 Wood Units in the absence of left heart disease, chronic lung disease or hypoxia, and chronic thromboembolic disease. PAH is an obliterative pulmonary arteriopathy that leads to morbidity and mortality, often due to right ventricular failure (RVF). Emerging evidence from preclinical research, using chemical inhibition or genetic depletion of inflammatory mediators, reveals a role for inflammation in the adverse pulmonary vascular remodelling in PAH. More recently, studies have also identified inflammation of the right ventricle (RV) as a potential contributor to RV decompensation and failure. While inflammation contributes to the pathogenesis of PAH, no approved PH-targeted therapies specifically target inflammation. Macrophages are myeloid cells that play a critical role in inflammation and PAH. Their cellular plasticity enables the acquisition of tissue-specific phenotypes and functions that may promote either resolution or exacerbation of inflammatory signalling. Macrophage plasticity in PAH is poorly understood. We examine how alterations in glucose metabolism, particularly the uncoupling of glycolysis from glucose oxidation—a notable feature of PAH observed in various cell populations—impact macrophage polarization and the inflammatory phenotype associated with PAH. The study of immune cell metabolism, known as immunometabolism, is an emerging field that has yet to be explored in PAH. Improving understanding of the inflammatory mechanisms in PAH, particularly novel pathways related to macrophage immunometabolism, may identify new targets for anti-inflammatory therapies for PAH.

## Introduction

Pulmonary hypertension (PH) is defined as an elevation of mean blood pressure in the pulmonary circulation above 20 mmHg, regardless of the cause [1]. Using this inclusive diagnostic criterion, over 1% of the world’s population has PH [1]. Within the broad syndrome of PH, there are five subgroups, defined by their aetiology, and this classification has therapeutic relevance. They include Group 1: pulmonary arterial hypertension (PAH), Group 2: pulmonary venous hypertension due to left heart disease, Group 3: pulmonary hypertension due to lung disease or hypoxemia, Group 4: chronic thromboembolic pulmonary hypertension, and Group 5: pulmonary hypertension due to diverse multifactorial mechanisms [1]. This review highlights the emerging area of immunometabolism in PAH. The occurrence of inflammation in PAH is well documented; however, the molecular basis of inflammation and its potential link to altered metabolism in inflammatory cells in PAH remains unclear.

Group 1 PH, or PAH, is an obliterative pulmonary vasculopathy primarily affecting small intrapulmonary arteries, and it is defined by a mean pulmonary arterial pressure (mPAP)>20 mmHg and pulmonary vascular resistance (PVR) ≥ 2 Wood Units in the absence of left heart disease, chronic lung disease or hypoxia, and chronic thromboembolic disease [2]. The pathogenesis of PAH is not fully understood, although it is clear that gene mutations, mostly autosomal dominant mutations of endothelial cell genes that are inherited with variable penetrance, epigenetic dysregulation, connective tissue diseases, impaired mitochondrial dynamics and metabolism, and exposure to drugs, such as amphetamines and anorexigens, and toxins, predispose to this syndrome [3]. PAH has an annual incidence of approximately six cases per million adults and a prevalence of 48–55 cases per million adults in middle- to high-income countries [2]. A population-based study of PH in Ontario, Canada identified 50,529 people with PH in the Institute for Clinical Evaluative Sciences (ICES) database between 2002 and 2012. This study noted that the incidence and prevalence of PH were increasing, with PAH accounting for 15.6% of all PH [4].

In PAH, the primary disease pathology is in small pulmonary arteries (PAs), and within these vessels, there is adverse remodelling in each layer of the vascular wall, including the intima (endothelial cells), the media (pulmonary arterial smooth muscle cells; PASMCs), and the adventitia (fibroblasts) [3]. Key features of PAH include vasoconstriction, endothelial dysfunction, excessive fibrosis, vascular stiffening, reduced vascular compliance, increased thrombosis and inflammation [3]. While vasoconstriction is a feature of PAH in some patients, structural remodelling of small PAs is the hallmark of PAH. Only 13.2% of patients challenged with acute vasodilators within the PAH biobank (a biobank including over 2800 subjects with PAH) positively responded to drugs, like inhaled nitric oxide, implying that obstructive vascular remodelling, rather than vasoconstriction, is the predominant cause of elevated PVR in PAH [5]. While endothelial cell injury is postulated to be an early trigger for PAH, ultimately, all cells in the pulmonary vasculature and right ventricle (RV) develop a pseudoneoplastic phenotype, meaning rates of proliferation or hypertrophy are increased, and/or unstimulated rates of apoptosis are decreased, reminiscent of the biology of cancer cells, reviewed in Mocumbi et al. [1]. Moreover, cells from patients with PAH develop mitochondrial metabolic changes reminiscent of those seen in cancer, with increased uncoupled glycolysis, known as the Warburg effect. These cells further present abnormal mitochondrial dynamics, with increased fission and impaired fusion [1]. These acquired mitochondrial metabolic abnormalities are recapitulated in animal models of PAH induced by monocrotaline (MCT) or the combination of exposure to SU5416, a VEGF-2 receptor antagonist, and hypoxia (SuHx) [6-8]. In the pulmonary vasculature, these acquired changes in mitochondrial metabolism and dynamics [9] favour accelerated cell proliferation, reduced mitochondria-mediated apoptosis and increased fibrosis [10], while in the RV, they favour cardiomyocyte hypertrophy and fibrosis [11].

The resulting vascular obliteration, vascular stiffening and vasoconstriction in small PAs increase RV afterload and resistance, measured as PVR [3]. This increase in afterload provokes RV hypertrophy (RVH), which is initially adaptive, meaning the RV maintains normal ejection fraction, cardiac output and filling pressures despite increased PVR; but over time, RVH often becomes maladaptive, manifesting as reduced RV ejection fraction, cardiac output and increased filling pressures with RV dilatation [12]. Eventually, most patients with PAH die related to RV decompensation and failure (RVF) [3]. Nonetheless, there is heterogeneity within PAH when considering predisposition to maladaptive RVH [3]. For example, those with Eisenmenger’s syndrome (PAH patients with PH due to congenital heart disease and intracardiac shunts, usually ventricular septal defects) are more likely to experience decades of adaptive RVH; conversely, patients with scleroderma (a connective tissue disease associated with inflammation and PAH) typically show maladaptive RVH and develop RVF earlier in the disease course and often develop RV failure at lower mPAP [3]. Interestingly, afterload is often higher in Eisenmenger’s syndrome than in scleroderma, indicating that afterload alone is not the sole determinant of RV decompensation or survival [3]. It is noteworthy that ~21% of all PAH occurs in patients with connective tissue diseases (CTD), like scleroderma; this is referred to as associated PAH (APAH-CTD) and is a subset of PAH in which a role for inflammation is well established [13]. This raises the possibility that RV failure is driven in part by RV inflammation, rather than solely by elevated RV afterload.

Over the past two decades, it has become clear that one hallmark of PAH is metabolic dysfunction related to acquired mitochondrial abnormalities, which affects most disease-relevant cells in the pulmonary vasculature and RV. The use of rodent models of PAH has allowed for the identification of altered metabolic pathways that include 1) altered fatty acid oxidation, variably reported to be either increased or decreased [14-16], 2) increased glutaminolysis [17], 3) activation of the pentose phosphate pathway [18,19] and 4) increased uncoupled glycolysis with decreased glucose oxidation [10,20-23], also known as the Warburg effect. The abnormalities in glucose metabolism are the most widely reported of these metabolic abnormalities in patients with PAH [24-27] and in patient-derived cells, such as PASMCs and endothelial cells [20].

The Warburg effect, initially described by Otto Warburg in 1927 in cancer cells [28,29], is a metabolic shift from glycolysis coupled with glucose oxidation (coupled glycolysis) to uncoupled aerobic glycolysis, in which glycolysis terminates in the cytosol, driving the production of lactate, rather than generating pyruvate to fuel oxidative metabolism in the mitochondria [3]. Diminished glycolytic coupling is linked to marked up-regulation of glycolysis, which maintains adenosine triphosphate (ATP) production despite the inhibition of glucose oxidation. However, uncoupled glycolysis has adverse effects on the quality control mechanisms and cell cycle regulation, inhibiting mitochondrial-mediated apoptosis and accelerating cell cycle progression, thereby promoting the survival of abnormal, hyperproliferative cells [30].

There exist multiple triggers for the Warburg effect, including epigenetic changes, such as up-regulation of DNA methyltransferases, leading to methylation of many genes, including mitochondrial superoxide dismutase 2 (SOD2) [31]. The epigenetic silencing of SOD2 leads to reductive alteration of redox state, which activates hypoxia-inducible factor 1α (HIF-1α) in normoxia, despite abundant oxygen availability [3]. This is a well-established mechanism of Warburg metabolism whereby normoxic activation of HIF-1α up-regulates the expression of pyruvate dehydrogenase kinase (PDK) and glucose transporters (GLUT). PDKs are a family of four enzyme isoforms that phosphorylate and inhibit pyruvate dehydrogenase (PDH) and thereby inhibit glucose oxidation [3]. Another pathway by which glucose oxidation can become uncoupled from glycolysis involves up-regulating a splice variant of the terminal enzyme in glycolysis, pyruvate kinase (PK). The increased expression of pyruvate kinase isozyme M2 isoform (PKM2), relative to the PKM1 isoform, occurs in PAH cells and promotes the Warburg effect [32]. Both of these metabolic changes relate to activation of HIF-1α, classically elevated in hypoxia, but also activated under conditions of both oxidant stress [33,34] and reductive stress [31]. HIF-1α not only up-regulates PDK expression [33] but also promotes the PKM isoform shift towards PKM2 [35]. PDK activation, PDH inhibition and PKM2 up-regulation occur in PAH and in many types of cancers [36,37].

Mutations of the bone morphogenetic protein receptor type II gene, *BMPR2*, are the leading cause of familial PAH and also occur in some patients with sporadic PAH [38,39]. Loss-of-function *BMPR2* mutations or BMPR2 down-regulation also lead to this Warburg phenotype and promote hyperproliferation of PAH PASMCs, fibroblasts and endothelial cells [3,38,39]. Thus, the Warburg effect may be triggered by gene mutations as well as by epigenetically mediated changes in cellular redox state. In PAH, the Warburg metabolic phenotype was first identified in PAH PASMCs [20], and subsequently, most disease-relevant cells were proven to manifest similar metabolic shifts, including PA endothelial cells [21], pulmonary adventitial fibroblasts [22], RV fibroblasts [10], and RV cardiomyocytes [23]. However, whether Warburg metabolism occurs in macrophages in PAH is still under investigation. Considering the importance of these cells in either promoting inflammation or maintaining organ homeostasis [40-42], understanding whether metabolism influences the function of macrophages has clinical relevance. Critically, understanding the immunometabolism of macrophages that belong to different niches (lung *vs*. RV), have distinctive origins (embryonic tissue *vs*. bone marrow) and exist in different activation states, becomes important, therapeutically.

Inflammation has been described in all forms of PH [43-47]. Inflammation is the innate immune response to harmful stimuli, such as infection or tissue damage, and can be cellular, humoral or both [48,49]. The inflammatory process involves both activation and resolution phases, and temporally, inflammation may be acute or chronic [50-52]. Several immune cells are involved in the process of inflammation in PAH, although the literature is controversial regarding the net contribution of specific types of immune cells in the pulmonary vasculature and RV to the pathogenesis of PAH, as reviewed in Mocumbi et al. [1].

## Inflammation in PAH

Inflammation has a significant role in PAH, whether it is perivascular inflammation, contributing to adverse pulmonary vascular remodelling [43,53,54] or RV inflammation, leading to RV dysfunction and RVF [40,42,55]. However, most approved drugs are vasodilators that do not directly address PAH’s inflammatory phenotype or obstructive vascular remodelling that are the hallmark of this syndrome. The exception to the vasodilator focus of PAH-targeted therapies is sotatercept, a newly approved drug which serves as a trap for activin and TGF-β superfamily members and may address aspects of adverse pulmonary vascular remodelling, accounting for sotatercept’s ability to reduce mPAP [56]. To the extent that PAH-targeted therapeutics promote vasodilation of pulmonary vessels and reduce mPAP and PVR, they partially counteract the increased RV afterload in this syndrome [3]. Importantly, approximately 28% of patients with PAH in the US-based PAH Biobank have APAH-CTD, which is inflammatory by nature [57], and even people with PAH but no connective tissue disease often have increased inflammation contributing to disease development and progression. Thus, the lack of approved anti-inflammatory therapies for patients with PAH is noteworthy and should be addressed, particularly as translational research has demonstrated substantial influence of inflammatory pathways on the pathophysiology of PAH [57]. Improving the understanding of the inflammatory mechanisms in PAH, particularly novel pathways related to immunometabolism, is valuable as it may identify new targets for anti-inflammatory therapies.

The functions of several types of immune cell are altered in PAH. These changes include a decrease in the number of anti-inflammatory regulatory T cells [58,59], an increase in inflammatory T helper cells [60,61], a decrease in natural killer cells [62] and an increase in macrophages [40,42]. However, this review focuses on the contribution of macrophages to the pathogenesis of PAH [5,40-42,53,63-71]. Macrophages contribute to disease progression primarily by secreting critical growth factors, such as platelet-derived growth factor-B (PDGF-β) [72], and inflammatory cytokines, such as interleukin-1 beta (IL-1β) [72,73]. These factors are involved in regulating tissue remodelling, including adverse RV remodelling [40,72,74-76]. Inflammatory cytokines relevant to PAH pathogenesis, such as IL-1β, IL-6, IL-8, IP-10, IL-12p40, IL-13 and IL-18, are elevated in the blood [5,75,77-81], lungs [82,83] and RV [40,74] of PAH patients, and levels of these cytokines correlate directly with disease severity and mortality [77,78,81].

We recently discovered that patients with a germline or somatic CHIP (clonal haematopoiesis of indeterminate potential) mutation in Tet methylcytosine dioxygenase 2 (*TET2*), a gene which encodes TET2, and a major enzyme mediating DNA demethylation, exhibit greater inflammation compared with those with PAH without such a mutation [5,84]. While metabolic dysregulation and immune cell activation occur in PAH, the connection between the cellular metabolism of immune cells in PAH and their inflammatory phenotype remains to be established. If changes in macrophage metabolism do regulate the macrophages’ inflammatory phenotype and cytokine production, this would represent an example of immunometabolism. Moreover, if the pro-inflammatory phenotype of macrophages in PAH were to be metabolically driven, this would suggest the possibility of reducing inflammation by targeting metabolic pathways inherent to immune cells or to the surrounding cells that make up the tissue microenvironment.

**Figure 1:**
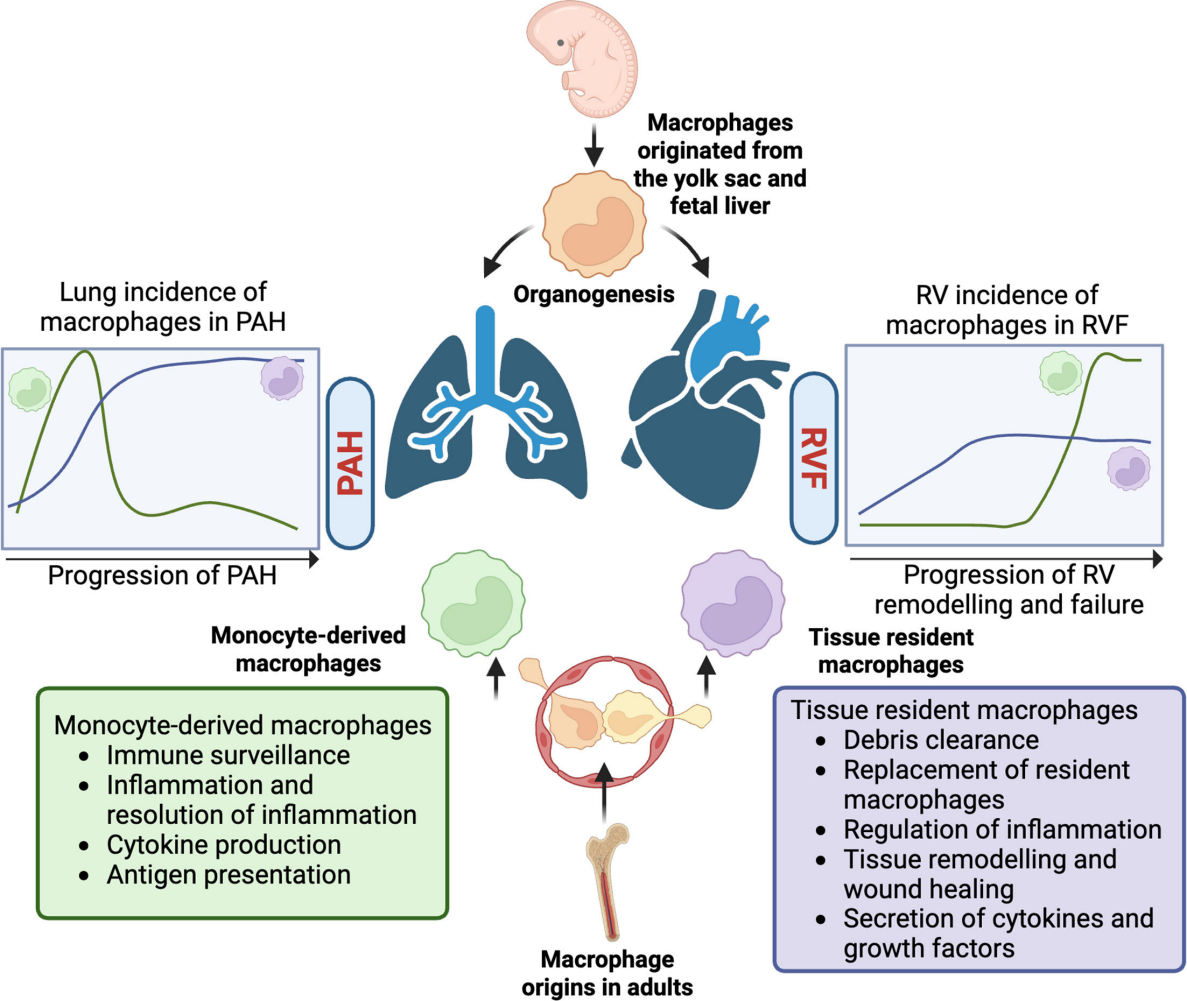
Macrophage origins and their phenotypes. Macrophages can originate from the yolk sac and fetal liver during embryogenesis or can be derived from bone-marrow from embryogenesis or can be derived from bone-marrow, creating subsets of tissue resident, self-renewing macrophages and subsets of circulating monocyte-derived macrophages, respectively. Circulating monocytes can enter tissues and differentiate into macrophages, which may have either pro-inflammatory or anti-inflammatory roles. Both tissue resident and bone marrow-derived macrophages are present in PAH, and their numbers and function differ over the time course of disease progression in both major organs affected, the lung and the right ventricle (RV). *Created with BioRender.com*

Macrophages are key players in inflammation and can be tissue-resident, meaning their presence in tissues is established during embryonic development, or they may migrate into tissues later in life, originating from circulating monocytes (Figure 1) [85]. This latter group of monocytes is bone marrow-derived myeloid cells which circulate in the blood before differentiating into macrophages within tissues [86]. Macrophages adopt different phenotypes and functions depending on the tissue in which they are located and the physiological state in which they exist. For example, in the lungs [87] and the heart [88], specific subsets of macrophages that are tissue-resident and self-renewing contribute to the normal development and homeostasis of these organs. With maturation or disease, monocyte-derived macrophages may migrate to the tissue to either replace resident macrophages or to resolve tissue injury (Figure 1) [89]. Both resident and monocyte-derived macrophages are further subclassified into subsets based on their immunophenotype and function. More importantly, macrophage subsets are tissue-specific and confer different immunological capabilities [90]. In the past, a somewhat simplistic classification of macrophages as inflammatory (also known as M1) or anti-inflammatory (also known as M2) was used; however, this nomenclature is outdated because it does not fully reflect the functional attributes of these cells *in vivo*, nor does it represent their tissue-specific plasticity. Therefore, rather than classifying macrophages as M1 *vs*. M2, we will use the terminology of resident *vs*. monocyte-derived macrophages to describe the cellular origin of these macrophages. Additionally, we will refer to their functions as inflammatory,indicating that these cells release inflammatory mediators upon the recognition of pathogens or disruption of the microenvironment; or anti-inflammatory, denoting their role in promoting tissue repair and remodelling (including fibrosis) through efferocytosis and the secretion of anti-inflammatory factors, as well as the implications within the tissue environment where applicable.

**Figure 2:**
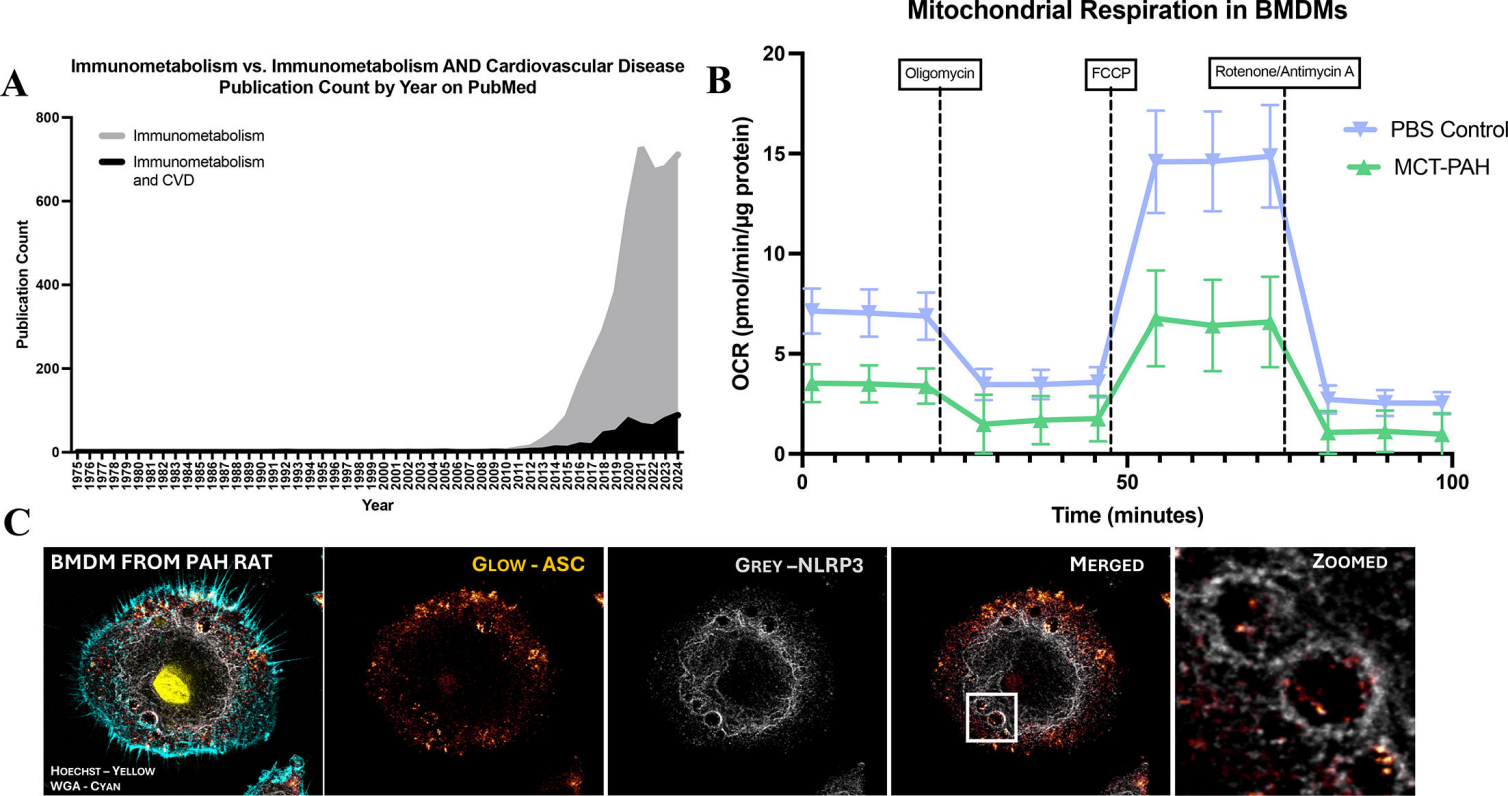
Immunometabolism is an emerging field of research with relevance to explaining the roles of macrophages in pulmonary arterial hypertension (PAH). (**A**) As of February 19, 2025, publication count per year including the search term ‘immunometabolism’ on PubMed, compared with the number of publications found using the search terms ‘immunometabolism AND cardiovascular disease’ (CVD). (**B**) Oxygen consumption is reduced in bone marrow-derived macrophages (BMDMs) from rats with monocrotaline-induced PAH (MCT) as compared with BMDMs from PBS-treated control rats (*n* = 1/group, three technical repeats/animal). (**C**) NLRP3 (NOD-like receptor protein 3) (grey) and ASC (apoptosis-associated speck-like protein containing a caspase activation and recruitment domain (CARD)) (glow), key proteins in the formation of the NLRP3 inflammasome, are present and interact in MCT-BMDMs, reflecting activation of this inflammatory pathway (*n* = 1).

Immunometabolism, the intersection of immunology and metabolism, is an emerging field of study that encompasses how immune cell metabolism regulates function. This is of scientific interest and has translational relevance, as it may identify novel metabolic therapeutic targets. A literature search using PubMed reveals that the term ‘immunometabolism’ was first used in 1975; however, since 2016, a rapid increase in its use has been observed, suggesting a growing interest in this field of study (Figure 2A). Alterations in the bioenergetics of immune cells, including macrophages, have been described in various diseases and conditions, including cancer [91], obesity [92], coronary artery disease [93], and infectious diseases [94]; however, immunometabolism is underexplored in PH.Only two studies have addressed the topic, with one examining the fibroblasts’ influence on macrophage metabolism in PH [95], and the other investigating the role of ferroptosis in endothelial cells in PAH, which causes metabolic changes in macrophages activation [96]. In the case of macrophages, glucose metabolism is particularly interesting as it is well-characterized that macrophages that utilize glycolysis as the primary source for ATP production have an inflammatory phenotype, whereas macrophages relying more on oxidative phosphorylation and fatty acid oxidation to generate ATP often exhibit anti-inflammatory properties [97,98].

Our group has been studying the glucose metabolism of macrophages in PAH. Since the Warburg effect is a phenomenon observed in many cell types in PAH, we are aiming to confirm whether uncoupled glycolysis also occurs in macrophages in PAH. Preliminary results suggest that macrophages, in this case, bone-marrow-derived macrophages (BMDM), from rats with PAH induced by the injection of MCT, are metabolically programmed to exhibit a reduced oxygen consumption rate (OCR) compared with macrophages from healthy animals (Figure 2B). Notably, BMDMs from MCT-PAH rats also express NOD-like receptor protein 3 (NLRP3) and ASC (apoptosis-associated speck-like protein containing a caspase activation and recruitment domain [CARD]) (Figure 2C), key proteins in the formation of the NLRP3 inflammasome. When NLRP3 and ASC interact, Caspase-1 is activated and subsequently cleaves IL-1β and IL-18 into their active forms. We previously showed an increase in monocyte-derived macrophages in the RV of patients and rats with PAH, which induces RVF, and noted activation of the NLRP3 inflammasome in these cells [40]. Moreover, inhibiting the NLRP3 inflammasome pathway, using MCC950, preserved RV function. While we did not study the immunometabolism profile of these RV macrophages, we showed that macrophages from MCT rats trigger mitochondrial fission in cardiomyocytes, and we speculated that this might contribute to the release of damage-associated molecular patterns (DAMPs), which are known to contribute to the recruitment of inflammatory monocytes to target tissues [40]. Ongoing research is investigating the content of inflammatory mediators in these cells, comparing healthy control- and PAH-BMDMs, and assessing whether targeting metabolism can prevent the release of pro-inflammatory cytokines such as IL-1β.

In this review, we focus on macrophages’ role(s) in PAH and summarize current knowledge on the immunometabolism of these cells, while highlighting important differences in macrophage polarization between the lungs *vs*. the RV in PAH. We also discuss the potential implications of macrophage metabolism in PAH as an underappreciated mechanism that may modulate their phenotype and function.

## Macrophages and glucose metabolism

Metabolism refers to diverse biochemical processes in mammalian cells that convert substrates, such as glucose and fatty acids, into molecules that feed the tricarboxylic acid (TCA) cycle and yield electron donors that fuel ATP generation by the mitochondrial electron transport chain (ETC) [99]. The predominant forms of metabolism in healthy adult mammalian cells are aerobic, meaning they depend on the availability of molecular oxygen as the terminal electron acceptor for the mitochondrial ETC. [99] In a healthy state, the cytosolic process of glycolysis, which generates 2 moles of ATP per mole of glucose, culminates in the generation of pyruvate, which is transported into the mitochondria via the pyruvate transporter. Within mitochondria, the process of oxidative phosphorylation generates a further 32 moles of ATP [99]. In PAH, glucose metabolism is altered such that there is increased aerobic uncoupled glycolysis in the cells of the pulmonary vasculature and RV [10,20-23]. This metabolic shift results from active suppression of either PDH activity (by PDK) or impaired pyruvate formation due to an isoform switch from PKM1 to PKM2 [1]. These metabolic changes alter cell proliferation and apoptosis in the pulmonary vasculature (favouring proliferation and impairing apoptosis) and, in RV cardiomyocytes, reduce contractility and promote hypertrophy [15,17,27]. Acquired metabolic abnormalities (PDK-mediated Warburg metabolism [10]) in RV fibroblasts also trigger fibroblast proliferation and collagen production [10]. Currently, the occurrence of altered glucose metabolism in macrophages in PAH, as well as the consequences of putative immunometabolic changes to the pathophysiology of PAH, requires further investigation.

**Figure 3:**
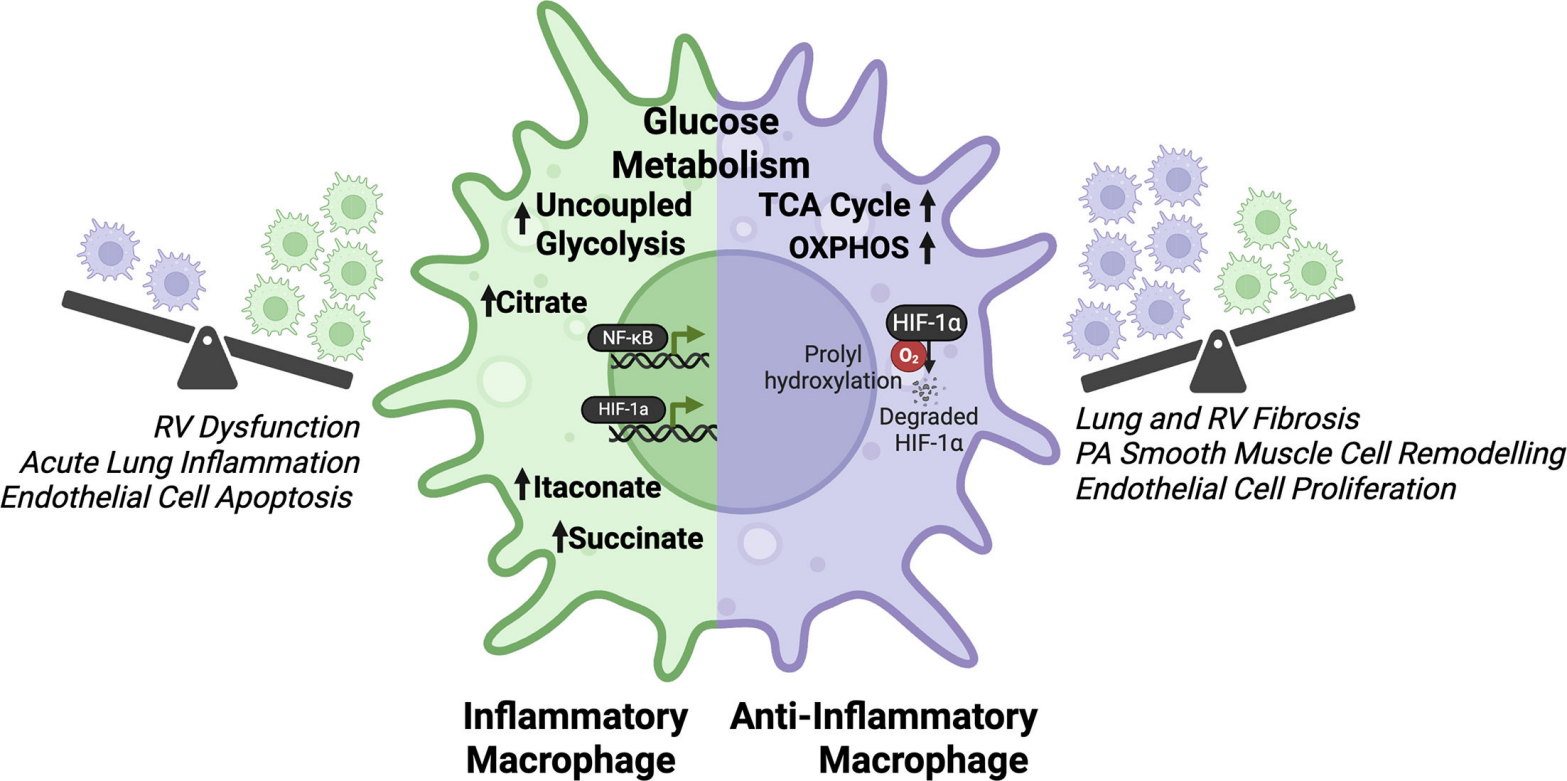
Differences in glucose metabolism in inflammatory *vs*. anti-inflammatory macrophages: implications for inflammation and the role of these macrophage subsets in the pathogenesis of P AH. Inflammatory macrophages are glycolytic and rely on Warburg metabolism, while anti-inflammatory macrophages rely on glucose oxidation. Both macrophage polarities are present in PAH, and each population has distinct roles in contributing to various aspects of disease progression. *Created with BioRender.com*

The metabolism of macrophages in healthy mice has been studied using BMDMs [98,100]. Functional *ex vivo* studies have shown that activating macrophages with either inflammatory factors, such as interferon-gamma (INF-γ) and toll-like receptor (TLR) agonists, or anti-inflammatory factors, such as IL-4 or IL-13, alters glucose metabolism towards either uncoupled or coupled glycolysis, respectively [98,101]. Notably, exposing BMDMs to conditioned media from fibroblasts obtained from PH animal models and humans also triggers a shift toward uncoupled glycolysis [95]. HHowever, whether metabolic changes can alter cytokine production and modulate macrophage plasticity remains a knowledge gap in the field. Predominantly, inflammatory macrophages display uncoupled glycolysis, while anti-inflammatory macrophages rely more on oxidative phosphorylation (Figure 3) [98,101]. The inflammation-associated metabolic shift towards increased glycolysis and reduced glucose oxidation in inflammatory macrophages is due to disruption of the TCA cycle at two ‘breakpoints’: 1) the conversion of isocitrate into α-ketoglutarate by isocitrate dehydrogenase (IDH) and 2) the conversion of succinate to fumarate by succinate dehydrogenase (SDH) [98]. The inflammatory consequence of TCA cycle inhibition at these two points is further compounded by HIF-1α and NFκB (nuclear factor-kappa B) up-regulation/activation and subsequent downstream effects’ (Figure 3) [102-104]*.*

HIF-1α-mediated increases in PDK1 expression promote an inflammatory polarization of macrophages, through an upstream shift in metabolism favouring uncoupled aerobic glycolysis [105]. Thus, PDKs may regulate a metabolic checkpoint in the polarization of macrophages towards the inflammatory phenotype [105]. Unlike PDK1, PDK2 and PDK4 are less studied. Min et al. found that knocking out PDK2/4 in mice blocks the shift toward an inflammatory phenotype in lipopolysaccharide (LPS) and INF-γ-stimulated macrophages. Macrophages from PDK2/4 knockout mice also have decreased activation of HIF-1α, along with a diminished Warburg metabolism and reduced levels of pro-inflammatory cytokines, including IL-1β, suggesting a potential positive feedback loop wherein HIF-1α increases PDK2/4 expression, which then further stimulates HIF-1α transcription [105]. PDK inhibitors, such as dichloroacetate (DCA) and KPLH1130, reduce the levels of pro-inflammatory cytokines and ameliorate mitochondrial function in mouse BMDMs, though KPLH1130 is a more potent inhibitor of PDKs, reducing pro-inflammatory cytokine levels at lower concentrations (5–10 µM) than DCA (0.5–2 mM) [105]. Additionally, KPLH1130 significantly reduced inducible nitric oxide synthase (iNOS), nitric oxide (NO) and HIF-1α levels, thereby reducing pro-inflammatory outputs while preventing the decrease of basal and maximal oxygen consumption rate caused by the metabolic reprogramming of mouse BMDMs towards an inflammatory phenotype [105].

**Figure 4:**
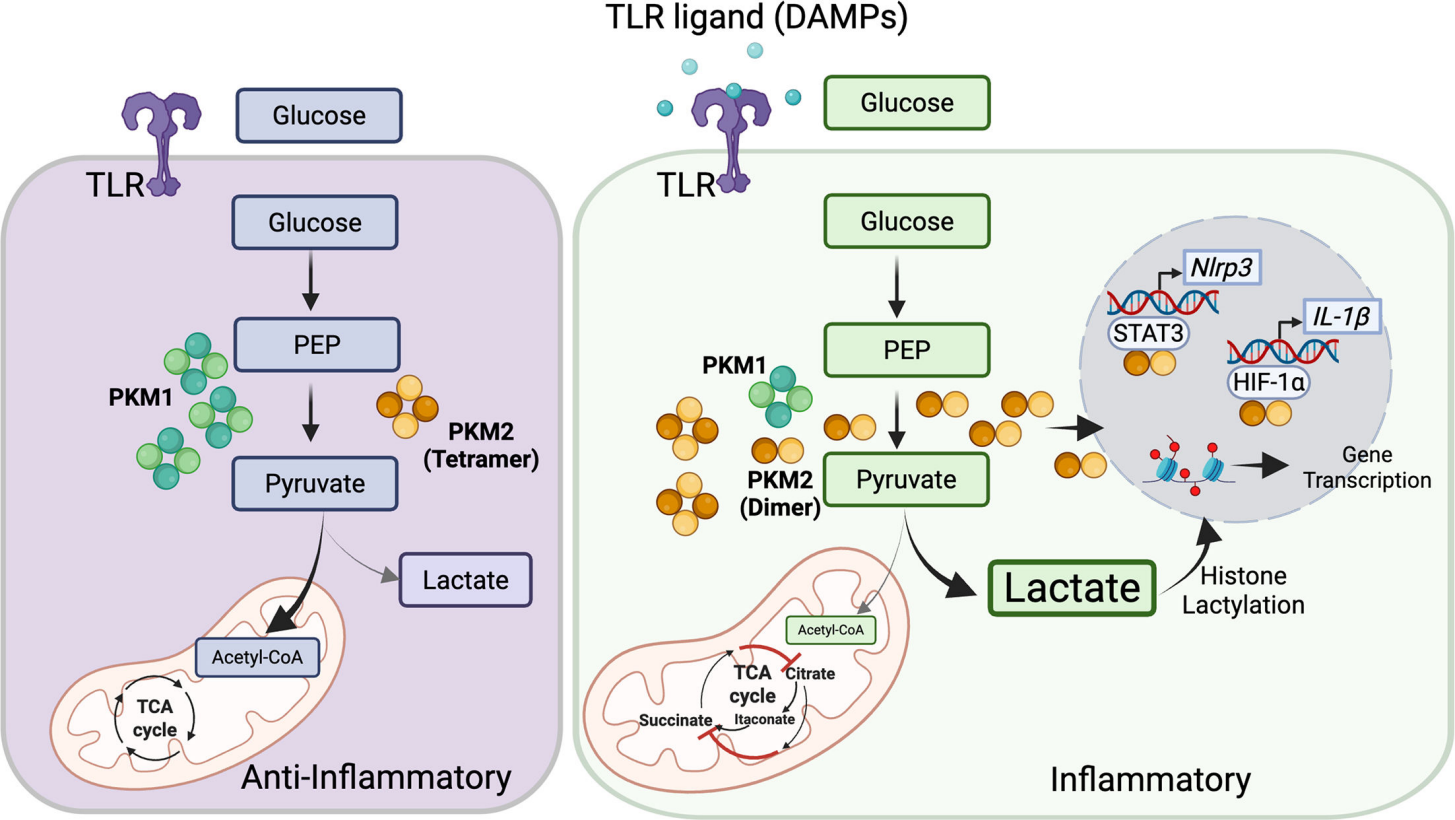
Pyruvate kinase muscle isozyme M2 (PKM2) is the potential link connecting Warburg metabolism with inflammatory phenotypes. In cells undergoing normal glucose oxidation, PKM1 is the primary isoform, while PKM2 is present in its high-activity tetrameric form, both of which favour glucose oxidation and the progression of pyruvate into the mitochondria, driving the TCA cycle. Macrophages express TLRs (toll-like receptors) that, upon activation by ligands such as DAMPs (damage-associated molecular patterns), trigger metabolic changes within these cells. In inflammatory macrophages, PKM2 is abundant in its less-active dimeric form, which favours the formation of lactate from pyruvate, therefore driving glycolysis. The PKM2 dimer can be translocated to the nucleus, where it functions as a co-activator of transcription factors by binding and stabilizing HIF-1α or through the phosphorylation of STAT3, thereby aiding in the transcription of the pro-inflammatory genes *Nlrp3* and *Il-1β*. Abundant cytosolic lactate in the cytosol of inflammatory macrophages, driven by the PKM2 dimer, leads to histone lactylation, which induces a time-dependent polarization by initially promoting transcription of inflammatory genes, following the initial rise in intracellular lactate, and subsequent activation of anti-inflammatory genes related to chronic exposure to TLR ligands. Thus, through multiple mechanisms, lactate production in inflammatory cells triggers shifts towards a more balanced phenotype. *Created with BioRender.com*

Along with PDKs, the expression and activity of PKMs play a significant role in regulating the inflammatory state of macrophages. Particularly, PKM1 and PKM2, responsible for converting phosphoenolpyruvic acid (PEP) to pyruvate, are rate-limiting enzymes in glycolysis. Both enzymes are encoded by the *Pkm* gene but are derived from alternative splicing [106]. While PKM1 is the predominant isoform in normal cells, the PKM2 isoform is up-regulated in cancer and PAH cells that exhibit the Warburg effect [107]. There is also an increased ratio of PKM2/PKM1 associated with Warburg metabolism in PA adventitial fibroblasts from both a PAH model (calves with severe PH) and samples from patients with IPAH, primarily driven by the down-regulation of PKM1 [22]. While PKM1 has high constitutive enzymatic activity (favouring pyruvate formation and the TCA cycle), PKM2 is less active and exists in its dimeric form. PKM2 can be allosterically activated into its tetrameric form by upstream glycolytic metabolites like fructose-1,6-bisphosphate, which, together with PKM1, enhance TCA cycle activity (Figure 4) [108]. Conversely, phosphorylation of PKM2 at Y105 by tyrosine kinases such as FGFR1, BCR-ABL and Jak2 prevents the formation of the active tetrameric form [109], thereby favouring glycolysis (Figure 4). In LPS-stimulated inflammatory macrophages, the expression of PKM2 is elevated, while its activity is reduced compared with unstimulated or IL-4-stimulated anti-inflammatory macrophages [110,111], suggesting a preference for glycolysis in inflammatory macrophages. As the less active dimeric form of PKM2 is predominant in LPS-activated macrophages, inducing tetramerization of PKM2 using either DASA-58 or TEPP-46, small molecules known to activate PKM2, reverses the LPS-induced Warburg effect while also inhibiting the expression of IL-1β [111]. This is consistent with the fact that increased activity of PKM2 paradoxically decreases lactate [112], as PKM2 can stimulate the production of oxaloacetate, which in turn inhibits lactate dehydrogenase A, the enzyme that generates lactate from pyruvate [112].

The PKM2–lactate relationship is particularly interesting, as it can drive the phenotypic plasticity of macrophages. In BMDMs stimulated by LPS, extracellular lactate activates PKM2 by post-translational modification, converting it from its low-activity dimeric form to a high-activity tetrameric state through lactylation. This process reduces its distribution in the nucleus and promotes an anti-inflammatory phenotype during chronic exposure to LPS [110]. When intracellular lactate is abundant, as seen in the Warburg effect, histone lactylation can also occur, with a significant impact on the macrophage phenotype. Macrophages exposed to LPS and IFN-γ show elevated intracellular lactate levels after 16 to 24 hours, which is temporarily associated with histone lactylation [113]. RNA sequencing and paired chromatin immunoprecipitation followed by sequencing (H3K18la, a specific histone lactylation site) indicated a time-dependent polarization of macrophages in response to LPS and IFN-γ (Figure 4). Inflammatory genes, such as *Nos2*, *Il6* and *Tnf,* are activated early, within as little as 4 hours post-stimulus, but show a steady decline at later intervals (8–24 hours). In contrast, anti-inflammatory genes such as *Arg1* and *Mmp9*, which are down-regulated at early time points (4 hours), show a significant increase in activity as H3K18la levels rise, a finding supported by protein analysis [113]. This shows that lactate production during inflammation can trigger a switch to a more balanced phenotype in the later phase.

Finally, PKM2 can also use non-canonical pathways to act as a protein kinase or transcriptional regulator, influencing macrophage phenotype and function. Nuclear, dimeric PKM2 can act as a co-activator for transcription factors by directly binding and stabilizing HIF-1α [111] to regulate the expression of inflammatory genes in macrophages [111]. PKM2 can also phosphorylate STAT3, strengthening the interaction between STAT3 and the *Mek5* promoter, a critical gene involved in cell proliferation in cancer cells [114]. Alternatively, PKM2 can enhance STAT3 phosphorylation and the binding of STAT3 to the *Nlrp3* promoter, leading to the transcription of this inflammatory gene in macrophages [115]. Indeed, macrophages stimulated with conditioned media from PH fibroblasts significantly increase mRNA levels of *Pkm2* and *Stat3*, along with other inflammatory genes (e.g. *Il1b, Hif1a, Il6 and Ccr2*) and glycolytic genes (*Glut1, Hk2 and Ldha*) [95]. These observations not only connect the Warburg effect to the inflammatory phenotype in these cells but also suggest a mechanism that is, in some models of PH, dependent on PKM2 activity (Figure 4).

## Inflammatory macrophages have breakpoints in their TCA cycles

**Figure 5:**
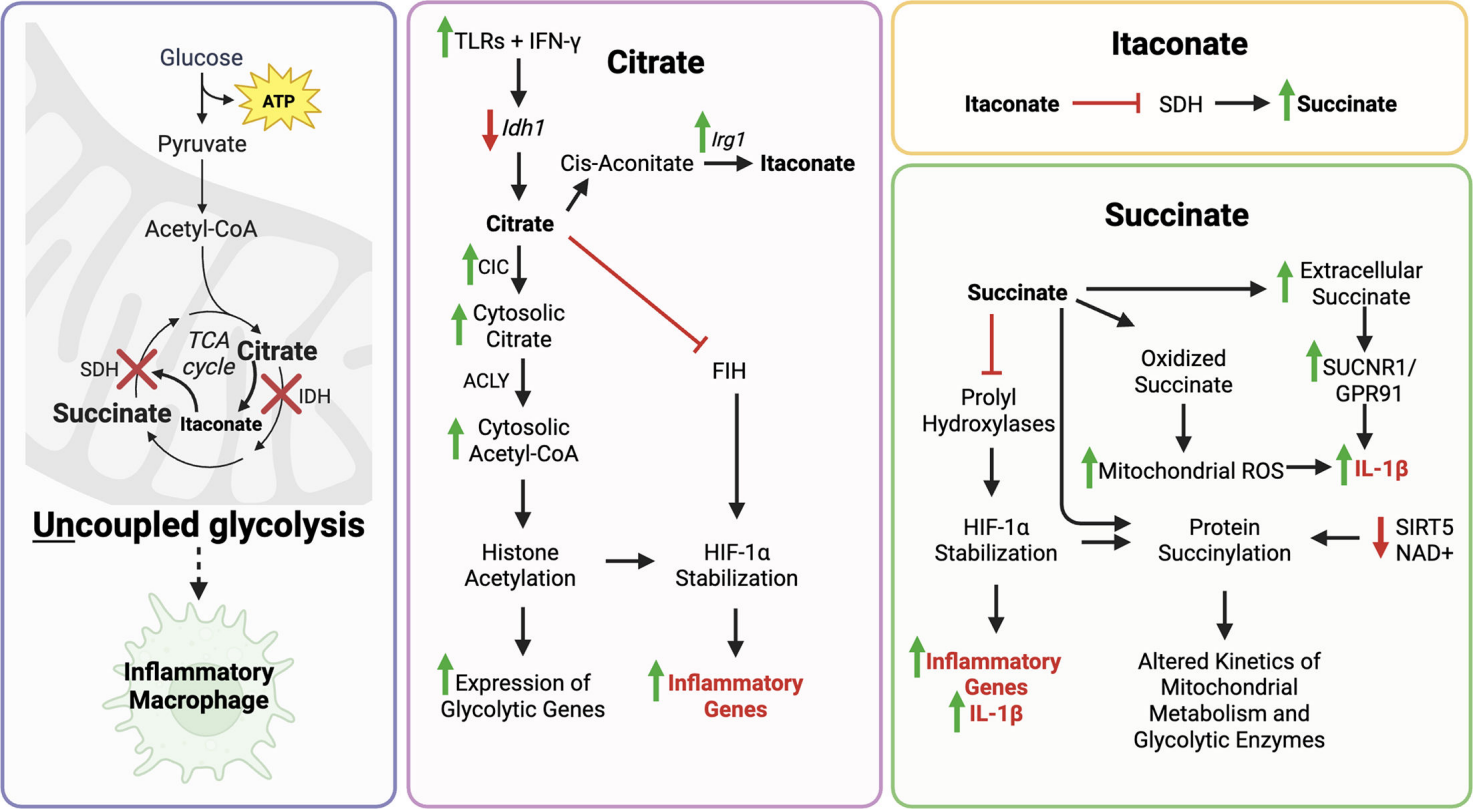
Changes in glucose metabolism promote uncoupled glycolysis in inflammatory macrophages. Uncoupled glycolysis in inflammatory macrophages is caused by two acquired breakpoints in the TCA cycle, causing a shift towards an inflammatory phenotype due in part to activation of HIF-1α. The accumulation of citrate, which is due to the down-regulation of isocitrate dehydrogenase (IDH), causes several downstream effects that lead to an increased proglycolytic and inflammatory genes, as well as the accumulation of itaconate. Itaconate inhibits succinate dehydrogenase (SDH), causing succinate to accumulate. The buildup of succinate has several downstream effects which increase expression of inflammatory genes, like *Il-1β*, and alter metabolism. ACLY, ATP-citrate lyase; CIC, mitochondrial citrate carrier; FIH, HIF asparaginyl hydroxylase; IFN-γ: Interferon-gamma; ROS, reactive oxygen species; TLR, toll-like receptor. *Created with BioRender.com*

Uncertainty exists regarding whether changes in metabolism precede and precipitate changes in inflammation or vice versa. It is also possible that metabolism within the macrophage and inflammation exists in a positive feedback loop. Most research on immunometabolism employs *in vitro* methods, activating macrophages with inflammatory or anti-inflammatory agents to study metabolic shifts. However, the impact of acquired metabolic changes within the macrophage on its function remains less explored. Recent studies indicate that blocking the TCA cycle at specific points leads to the buildup of metabolites like citrate, itaconate and succinate, which could enhance inflammation(Figure 5) [98]. The TCA cycle, critical for glucose oxidation, occurs within the mitochondrial matrix [116]. There, pyruvate derived from glycolysis is converted into acetyl-Coenzyme A (CoA) before entering the TCA cycle, where it is used to generate the reducing equivalents nicotinamide adenine dinucleotide (NAD^+^/NADH) and flavin adenine dinucleotide (FAD/FADH_2_). These electron donors ultimately transfer electrons to the mitochondrial ETC. fueling electron flux and promoting membrane potential generation across the outer mitochondrial membrane, which drives ATP synthase and generates ATP [116].

Inflammatory macrophages accumulate citrate due to the inhibition of the TCA cycle at IDH, the enzyme that catalyzes isocitrate into α-ketoglutarate. Similarly, metabolomic analysis utilizing liquid chromatography-mass spectrometry demonstrates that BMDMs exposed to conditioned media from PH fibroblasts also accumulate citrate, malate and fumarate, indicating alterations in the TCA cycle occur in response to the ‘PH environment’ [95]. In inflammatory macrophages, *Idh1* is transcriptionally down-regulated via the upstream activation of TLRs by LPS and IFN-γ [98]. The expression of the mitochondrial citrate carrier (CIC), which transports citrate from the mitochondrial matrix into the cytosol, is increased in LPS-stimulated macrophages at the mRNA and protein levels [117] and is also up-regulated in response to the pro-inflammatory cytokines tumour necrosis factor alpha (TNF-α) and IFN-γ [118]. When citrate accumulates in the cytosol, it can be cleaved into acetyl-CoA and oxaloacetate by ATP-citrate lyase (ACLY) [119]. The maintenance of acetyl-CoA in the cytosol is particularly relevant due to its role in histone acetylation, including histones H3 and H4 [119]. Histone acetylation, which increases in proportion to cytosolic acetyl-CoA, enables the positive regulation of genes involved in glycolysis, such as the glucose transporter GLUT4, hexokinase 2 (HK2), phosphofructokinase-1 (PKF-1) and lactate dehydrogenase A (LDH-A) [120]. Additionally, citrate negatively regulates HIF asparaginyl hydroxylase (FIH), which inhibits HIF-1α [121]. The negative regulation of FIH stabilizes HIF-1α, thereby increasing its transcriptional activity [102]. Citrate accumulation in the cytosol has a potential role in up-regulating HIF-1α targeted genes, including those encoding vascular endothelial growth factor (VEGF), erythropoietin (Epo), glucose transporters and glycolytic enzymes [102]. Therefore, citrate accumulation is predicted to reinforce Warburg metabolism and inflammation.

The accumulation of itaconate, generated from citrate-derived aconitate, has also been reported in inflammatory macrophages [98]. Itaconate accumulates due to the up-regulation of *Irg1*, which encodes aconitate decarboxylase, ACOD1. This enzyme catalyzes the decarboxylation of cis-aconitate into itaconate [122]. In a study using an integrated metabolic-transcriptional profiling approach, Jha et al. have shown that *Irg1* is one of the most up-regulated transcripts in inflammatory macrophages (compared with undifferentiated macrophages) [98]. In contrast, itaconate can also be considered anti-fibrotic in some diseases, as in idiopathic pulmonary fibrosis, where patients exhibit decreased itaconate and specifically a decrease in ACOD1 in alveolar, tissue-resident macrophages [123]. This is suggested to contribute towards a more anti-inflammatory and pro-fibrotic phenotype [123], which is also relevant in the development of fibrosis in PAH in the later stages of the disease (Figure 3) [123]. Subsequently, itaconate drives another breakpoint in the TCA cycle, as it inhibits SDH levels and activity (Figure 5) [124]. SDH catalyzes the oxidation of succinate to fumarate, and its inhibition results in the accumulation of succinate with downstream consequences that contribute to the inflammatory phenotype and Warburg metabolism [124].

In LPS-stimulated BMDMs, the accumulation of succinate inhibits prolyl hydroxylases [103] and oxidized succinate generates mitochondrial ROS (reactive oxygen species) [125], thereby stabilizing HIF-1∝ and favouring an inflammatory phenotype (Figure 5) [103]. This metabolic change creates a state of ‘pseudohypoxia’ where, despite abundant oxygen, HIF-1α is activated [103,126]. HIF-1α, once stabilized and activated, up-regulates the gene expression of the pro-inflammatory cytokine IL-1β, while also regulating expression of other HIF-1α target genes, including activating transcription factor 3, lysyl oxidase and ankyrin repeat domain 37 [103]. These downstream effects, along with the accumulation of succinate, also trigger protein succinylation [103], a post-translational modification that switches the protein charge from positive to negative [127]. The shift in charge by protein succinylation can alter the kinetics of enzymes involved in mitochondrial metabolism (Figure 5) [127,128]. In LPS-stimulated macrophages, when succinate accumulates, the expression of the desuccinylase *Sirt5* and NAD+is inhibited [103,129]. While this is evidence for enhanced glycolysis and lower respiration, it also suggests that protein succinylation results from increased succinate and decreased expression and activity of SIRT5 [103,129]. The reduced desuccinylase activity increases the succinylation of several glycolytic proteins and enzymes, including malate dehydrogenase, glutamate carrier 1, glyceraldehyde-3-phosphate dehydrogenase, L-lactate dehydrogenase A chain and transaldolase [103]. Further, the succinate receptor (SUCNR1/GPR91), expressed in several tissues [130], is up-regulated in inflammatory macrophages [131]. Succinate is also released in the extracellular space after necrosis or pathological damage [132] and can trigger autocrine and paracrine signalling via SUCNR1/GPR91, culminating in increased IL-1β release [131].

Therefore, the accumulation of TCA metabolites, specifically citrate, itaconate and succinate, merits further study in inflammatory diseases, particularly in the context of PAH, and represents a potential pro-inflammatory mechanism of immunoregulation. The accumulation of these metabolic intermediaries not only triggers changes in inflammation, both through activation of HIF-1α and post-translational modification of numerous enzymes but may also have value as biomarkers of inflammation.

## Macrophages in pulmonary vascular remodelling in PAH

Two main types of macrophages are present in the lungs in health: alveolar macrophages and interstitial macrophages. Alveolar macrophages are resident cells originating from embryonic development, whereas interstitial macrophages have more diverse origins. Interstitial macrophages can develop during embryogenesis and possess the ability to renew themselves, or they can be recruited from circulating monocytes [133]. Based on the expression of surface markers, alveolar macrophages are distinguished in humans as CD11b^+^HLA-DR^++^CD206^++^CD169^+^ [134] and in mice as Siglec F^+^CD11c^+^CD64^+^F4/80^+^CD11b^-^ [135]. Distinctively, interstitial macrophages are defined as CD11b^+^HLA-DR^++^CD206^+^CD169^-^ in humans [134] and CD11b^+^CD11c^+^CD64^high^MHCII^+^CD24^-^ in mice [135]. In PAH, extensive work has been done to understand the role of lung macrophages in disease pathophysiology [5,40,41,63-68]. Perivascular inflammation correlates with adverse vascular remodelling and trends to be increased in patients with more severe elevation of mPAP [43]. Florentin and colleagues demonstrated that in humans, interstitial macrophages (CD206^+^CD14^+^CD169^int^) are increased in the lungs of PAH patients, while alveolar macrophages (CD206^+^CD14^int^CD169^+^) are decreased [41]. Another study including 26 PAH patients with congenital cardiac shunts showed that recently-recruited macrophages (defined as MAC387^+^ cells) were elevated in the peripheral PAs and were more abundant in a subgroup of PAH patients with more advanced histological lesions, though the specific phenotype and metabolism of these macrophages remain unknown [63].

Investigating the functions and phenotype of macrophages in human lungs affected by PAH is challenging due to the invasiveness of sampling, specifically the relative paucity of human lung tissue for study; therefore, experimental models of PAH are important surrogates. However, it is important to acknowledge the limitations of these experimental models. Rat models are preferred for studying PAH due to their size (which permits haemodynamic profiling) and the similarities in the pathophysiology of PAH compared with humans. However, the availability of markers to identify different subsets of macrophages and their functions in rats is significantly more limited than in mice and humans, making it challenging to fully characterize these cells. On the other hand, while wildtype mice do not develop severe PAH (unlike rats), the molecular precision of mouse models, especially when considering genetically modified strains, makes them a valuable tool for discovering functionally relevant molecules related to PAH.

The chronic hypoxia model in mice is recognized as the gold standard for investigating pulmonary inflammation and remodelling in PH. In this mouse model, which is a model of Group 3 PH (not PAH), alveolar and interstitial macrophages within the lungs undergo dynamic changes over time, and their occurrence is associated with disease progression (Figure 1) [136]. In a time-course study by Pugliese et al., the alveolar macrophages maintained an inflammatory profile throughout exposure to chronic hypoxia for up to 28 days, while interstitial macrophages shifted towards an anti-inflammatory and pro-fibrotic phenotype between days 4 and 14 [136]. This contrast was especially noticeable 14 days after exposure to hypoxia [136]. Another study found four subsets of interstitial macrophages (also called intramural macrophages), where the prevalence of each subpopulation changed dramatically as hypoxic PH progressed [137]. These authors identified the transcriptional profile that characterizes the function and activation state of each macrophage subset, though their metabolic states were not studied. These subsets are: 1) MHCII^hi^CCR2^+^EAR^-^ (pro-homeostatic), 2) MHCII^hi^CCR2^+^EAR^+^ (pro-inflammatory), 3) VCAM^hi^TLF^+^ (as expressing *Timd4*, *Lyve1* and *Folr;* anti-inflammatory) and 4) VCAM^lo^TLF^+^ (pro-homeostatic) [137]. The authors named the subsets MHCII^hi^CCR2^+^EAR^-^ and VCAM^lo^ TLF^+^ pro-homeostatic because these cells were highly enriched with transcripts related to cellular homeostasis, including cell cycle regulatory genes, suggesting a tissue maintenance function [137]. The subset MHCII^hi^CCR2^+^EAR^+^ was called pro-inflammatory because they were enriched with pathways related to hypoxia and inflammatory response, such as IL2/STAT5 signalling, IL6/Jak signalling and IFN-γ response [137]. Finally, the subset TLF^+^VCAM^hi^ was called anti-inflammatory because it was enriched with genes related to TNFα signalling via NFκB, Hedgehog signalling and Notch signalling pathways associated with vascular remodelling [137-140]. When examining the effects of hypoxia over time, the proportions of the pro-homeostatic and anti-inflammatory subsets initially decrease early in hypoxia (reaching their lowest levels by day 3 post-hypoxia). While the pro-homeostatic macrophages return to levels comparable with those in healthy mice by day 21, the anti-inflammatory TLF^+^VCAM^hi^ macrophages reach peak frequency at day 21, exceeding the levels observed in healthy animals by approximately 10% [137]. In contrast, the number of pro-inflammatory MHCII^hi^CCR2^+^EAR^+^ macrophages increases as early as 1 day post-hypoxia, reaching their maximum at day 3 [137]. The number of these macrophages decreases in the following days, returning to normal levels by day 21 [137]. Taken together, the diverse subsets of macrophages are regulated over time and reflect the onset and progression of PH (Figure 1). Initially, an acute inflammatory response is driven by inflammatory macrophages. As hypoxia is sustained, an anti-inflammatory process, driven by the anti-inflammatory macrophages, is initiated to contain the damage. Persistently high levels of anti-inflammatory macrophages with chronic hypoxia, however, favour adverse effects such as vascular remodelling and fibrosis. It is unknown if similar changes in macrophage populations occur in patients with Group 1 PH.

MCT causes an inflammatory form of PAH in rats but is ineffective in mice. Unfortunately, while the PAH is severe in rats exposed to MCT, the marker sets to sort and identify subsets of lung macrophages in rats are less defined than in mice and humans. In PAH rats induced by MCT, inflammatory macrophages are identified, *in situ,* as CD68^+^NOS2^+^ [70]. These cells increase in number within the lung tissue by day 3 after MCT, persist at elevated numbers until day 7, and then drastically decrease in number by day 14 [70]. In contrast, anti-inflammatory macrophages (CD68^+^CD206^+^) begin accumulating on day 7 post-MCT injection, with the accumulation continuing until day 28 after MCT [70]. Given that pulmonary hypertension in MCT-PAH typically becomes haemodynamically apparent around day 10 post-injection, it is reasonable to consider changes in the lung’s immunological environment before the haemodynamic disturbance occurs. Consistent with this, Al-Qazazi and Lima et al. identified increased numbers of anti-inflammatory macrophages, identified as CD68^+^ expressing Arginase 1 and CD163 in the lungs of MCT-PAH rats on day 24, when PAH was well established [40].

Single-cell sequencing of lung cells from two rat models of PAH (MCT and SuHx) showed that macrophages are the primary cells affected by the disease [141]. However, the analysis underscored significant differences between the two models, which may be linked to variations in their pathophysiology. While in MCT-PAH, interstitial macrophages are drastically increased compared with control rats (>90% increase); in PAH rats induced by SuHx, alveolar macrophages are the cells primarily regulated [141]. This group further demonstrated that alveolar macrophages and non-classical monocytes (usually anti-inflammatory) are the cells with the strongest transcriptomic shift in MCT and SuHx rats, as detected by Differentially Expressed Genes (DEGs) analysis [141]. Notably, the typical pathways activated in both alveolar and interstitial macrophages across both rat models reflected TNFα signalling through NFκB and HIF pathways [141]. Pertinent to this review, Hong et al. noted in MCT rats that genes associated with oxidative phosphorylation are more up-regulated than those related to glycolysis in alveolar and interstitial macrophages, indicating a more anti-inflammatory phenotype in MCT lung macrophages [141]. This finding supports the notion that oxidative metabolism is linked to the anti-inflammatory and pro-fibrotic macrophage phenotype observed in the lungs of PAH patients [141]. Conversely, in SuHx rats, the same genes associated with oxidative phosphorylation are down-regulated in alveolar interstitial macrophages [141]. These differences in macrophage profiles between MCT and SuHx models could offer valuable insights into the specificity of therapies targeting molecular immunomodulatory pathways in patients with PAH, which may vary among individuals and be regulated in a case-specific manner.

Functionally, macrophages contribute to pulmonary inflammation and remodelling associated with PAH. *In vitro* studies suggest that macrophages play a role in regulating endothelial cell apoptosis and proliferation in PAH. Inflammatory macrophages co-cultured with healthy endothelial cells (human umbilical vein endothelial cells; HUVECs) trigger apoptosis in these cells, while the apoptosis rate is reduced when endothelial cells are co-cultured with anti-inflammatory macrophages [70]. Supernatants from anti-inflammatory macrophages promoted the proliferation of endothelial cells and PASMCs [70], increasing the percentage of cells in S and G2/M phases of the cell cycle in both cell types [70]. This indicates that in the later stages of inflammation, where anti-inflammatory macrophages are abundant and vascular remodelling occurs, macrophages promote the proliferation of both endothelial cells and PASMCs [70]. Indeed, Abid and colleagues (2018), using animal models of PH and PH human samples, demonstrated the importance of the crosstalk between PASMCs and macrophages via the chemokine receptors CCR2 (chemokine receptor 2) and CCR5 (chemokine receptor 5), as an essential mechanism affecting both cell types, and determining the migration and proliferation of PASMCs during the development and progression of PAH [142].

Lung macrophages also contribute to the extracellular matrix (ECM) remodelling in PAH. Inflammatory macrophages increase vascular stiffness by secreting matrix metalloproteinases (MMPs), which modulate and enhance ECM breakdown and collagen deposition [143]. MMP-10 is produced by human inflammatory macrophages *in vitro*, and its expression is increased in lung tissue and serum of PAH patients and in the lungs of MCT-PAH rats [144]. MMP-10 overexpression in inflammatory macrophages promotes PASMC proliferation and migration, contributing to adverse vascular remodelling [144]. Validating the role of macrophages in ECM remodelling, the depletion of monocyte-derived macrophages, using either clodronate-liposomes or gadolinium chloride, prevented the adverse remodelling of the pulmonary vascular ECM and mitigated pulmonary hypertension in PH rats exposed to chronic hypoxia [145]. This further highlights the role of macrophages as regulators of ECM remodelling and identifies their contribution to early disease progression.

## Cardiac macrophages in the RV in PAH

The diversity of cardiac macrophages in the left ventricle (LV) of both murine and human hearts has been the subject of extensive research. The field has progressed, leading to a deeper understanding of macrophage phenotypes in healthy hearts and the disease-specific changes that occur in the LV [146]. In contrast, the immunological niche of macrophages in the RV in PAH has received significantly less attention. This is partially due to the limitations of studying RV macrophages in humans and the challenges of using rat models of PAH, as available markers for characterizing macrophages in rats are restricted (relative to the broad marker sets available for murine research) [147]. Nonetheless, our group identified at least two macrophage subsets residing in the RV of two PAH rat models with evidence of RV decompensation (MCT and SuHx). These macrophage subsets were distinguished based on the expression of CCR2 in CD68^+^ cells as CCR2^+^CD68^+^ and CCR2^-^CD68^+^ [40]. CCR2^+^ indicates the cells are presumptively monocyte-derived and thus migrated from the bloodstream to the RV. In contrast, CCR2^-^CD68^+^ macrophages are believed to represent resident macrophages [40].

Further, our group studied the role of RV macrophages in the transition from adaptive remodelling to maladaptive RVH [40]. Using different rat models of PAH with adaptive RVH (created by PA banding) and maladaptive RV remodelling (created by using MCT or SuHx), we detected an RV-specific increase in the number of monocyte-derived macrophages in rats with maladaptive RV remodelling and RVF [40]. This monocyte-derived macrophage population (CCR2^+^CD68^+^) was less abundant in the RVs from the animals with adaptive RVH [40]. Indeed, the number of RV macrophages was inversely correlated with cardiac output during the maladaptive phase of MCT-PAH (i.e. during the transition from compensated to decompensated RV remodelling, which occurs between weeks 3 and 4) [40]. The observation that these RV macrophages expressed CCR2 suggests they originated as monocytes and migrated into the RV in response to a homing signal. Our group is currently conducting a time-course experiment using MCT-PAH rats. The goal is to track the dynamic changes in the incidence of resident CCR2^-^ and monocyte-derived CCR2^+^ macrophages in the RV tissue throughout the progression of PAH, particularly during the transition from compensated to decompensated RV remodelling. Unpublished data suggest that both macrophage subsets, resident CCR2^-^ and monocyte-derived CCR2^+^, increase starting at day 14 after MCT administration before RV dysfunction occurs, with CCR2^+^ macrophages playing a more significant role during the later transition to RV decompensation (Figure 1). This observation suggests that RVF is not solely a response to increased RV afterload; rather, it presumptively identifies RV inflammation as a precipitant of RVF.

The RV macrophages in PAH express increased levels of NLRP3, a key molecule involved in forming the NLRP3 inflammasome. Inflammasomes are multiprotein complexes, which include an inflammasome sensor molecule, the central inflammasome adaptor ASC, and Caspase-1 [148]. Inflammasomes are triggered as part of the innate immune signalling when membrane receptors or cytoplasmic sensors identify DAMPs, including metabolites generated due to metabolic imbalance. Once DAMPs are detected, NLRP3 and ASC interact, resulting in the activation of Caspase-1 that subsequently cleaves the pro-cytokines IL-1β and IL-18 into their active form, which are released from the cell [71,148]. Inflammasome activation will also cause pyroptosis, a rapid, Caspase-1 dependent form of programmed cell death [148]. Our group was the first to report the increased expression of NLRP3 in RV macrophages from rats and patients with PAH and to establish a relationship between this mechanism of inflammation and RVF [40]. Crucially, although NLRP3 was mainly found in RV macrophages, rather than in other cardiac cells [40], it is still unclear which RV macrophage type (CCR2^+^ *vs.* CCR2^-^ or both) exhibits the highest activity of the NLRP3 inflammasome pathway in PAH.

The protective effect of pharmacological inhibition of the NLRP3 inflammasome on RV function in experimental PAH models suggests that inflammation indeed contributes to RVF. We showed that the NLRP3 inhibitor, MCC950, improves measures of RV function, including tricuspid annular plane systolic excursion (TAPSE), cardiac output and ventricular-to-arterial coupling, in rats with MCT-PAH [40]. MCC950 also reduces pulmonary hypertension, as evidenced by the prolongation of the pulmonary artery acceleration time (PAAT) and a reduction in the right ventricular systolic pressure (RVSP), as measured on right heart catheterization (RHC). Finally, MCC950 caused beneficial PA remodelling, reducing pulmonary arterial medial wall thickness and also tending to reduce RVH [40]. MCC950 treatment also reduced the expression of RV NLRP3 and cleaved IL-1β in the RV [40]. Interestingly, MCC950 did not reduce the number of RV macrophages, suggesting that it was the activation of the inflammasome, rather than the presence of RV macrophages, that was harmful [40].

The contribution of macrophages to RVF in PAH was further investigated by treating MCT-PAH rats with the GP130 antagonist, SC144. SC144 prevents the activation of the IL-6 family signalling pathway (via STAT3 phosphorylation), which is important for macrophage proliferation and recruitment [149]. Unlike MCC950, SC144 reduced RV macrophage number, accompanied by a reduction in STAT3 phosphorylation and NLRP3 expression in macrophages [40]. Coincident with reduced numbers of activated RV macrophages in SC144-treated rats, cardiac function improved, evident as increased TAPSE, cardiac output and ventricular-to-arterial coupling [40]. The benefits of SC144 in cardiac function, however, occurred independent of any change in PVR or pulmonary vascular remodelling [40]. Thus, it is reasonable to speculate that inhibiting the migration of monocyte-derived macrophages to the RV during the development of PAH without disrupting resident macrophages might have a therapeutic benefit. Consistent with this, depletion of macrophages using clodronate liposomes in the SuHx PAH-rat model, post-disease establishment, improved RV function associated with ECM composition, evidenced by a reduced content of collagen I, III and IV, and other glycoproteins [150]. Thus, inhibition of the inflammasome or prevention of migration of blood monocytes to the RV, where they become inflammatory macrophages, appears beneficial in regressing experimental PAH. In translational studies using human RV PAH tissues, several domains of this pathway appear active in humans with PAH and RVF [40]. However, while these studies attest to the pathogenic role of monocyte-derived macrophages in PAH, the metabolic status of these immune cells was not measured, and thus, the role of immunometabolism was not addressed.

In healthy mice, most cardiac macrophages originate from embryonic (yolk sac) and fetal monocytes (liver), with a small subset derived from circulating monocytes (haematopoietic origin) [88]. Genetic fate mapping analysis using the lineage marker Flt3 (FMS-like tyrosine kinase 3; CD135), an essential kinase in the normal development of haematopoietic stem cells, confirmed that most cardiac macrophages in a non-diseased state are Flt3^-^ cells (>85%; also, CCR2^-^ and Ly6C^-^) meaning they are arriving in the heart during embryogenesis and are maintained throughout a lifetime via self-renewal [88]. Further phenotypic stratification of cardiac macrophages from the left ventricle shows that at least four subsets of macrophages can be identified: [1] MHCII^hi^CCR2^-^Ly6C^-^ [2], MHCII^lo^CCR2^-^Ly6C^-^ [3], MHCII^+^CCR2^-^Ly6C^+^ and the monocyte-derived [4] Ly6C^+^CCR2^+^ [88]. In pathological conditions such as systemic hypertension, the circulating monocyte-derived macrophage subset seems to mediate cardiac inflammation, as demonstrated in mouse models of hypertension [133,151]. Single-cell sequencing of the hearts of five-month-old neonatal mice *vs*. adult mice with end-stage left heart failure confirmed that all four subsets of cardiac macrophages exist in the heart, but they are different in abundance from those in healthy animals [151]. During cardiac injury, in both mouse and human, the migration of monocyte-derived macrophages (Ly6C^+^CCR2^+^) to the heart contributes to the activation of inflammatory pathways, including the NLRP3 inflammasome, while the self-renewal of resident macrophages limits adverse tissue remodelling [133,151]. In contrast with this LV-relevant data, the diversity of RV macrophages in the context of PAH is still unknown [151].

While some macrophage populations may be harmful and contribute to disease progression, others are beneficial. Thus, non-specific depletion of all macrophages is unlikely to be a therapeutic strategy for most cardiovascular diseases. Indeed, in systemic hypertensive rats, global depletion of macrophages, achieved using clodronate, worsens cardiac function but improves fibrosis, suggesting that some macrophages may have both protective and pathological functions [152]. This observation suggests that different macrophage subsets with specific functions must exist. For example, selective depletion of resident macrophages in systemically hypertensive Cx3cr1^CreERT2^, ROSA26^TD/DTR^ mice [151] has demonstrated that the lack of these resident macrophages prevents adaptive remodelling during cardiac injury from systemic hypertension, thereby worsening cardiac dysfunction in the presence of chronic systemic hypertensive stress [151]. The depletion of macrophages in multiple organs using diphtheria toxin (CD169^DTR^ mice), including the heart, caused diastolic dysfunction (reduced ventricular volume, early *vs*. late diastolic transmitral valve passive *vs*. active velocity wave peak velocities (E/A) ratio) and systolic dysfunction (reduced cardiac output) without systemic inflammation or non-cardiac organ damage [153]. Furthermore, the depletion of macrophages, followed by the recovery of these cells, restores LV function, as indicated by the recovery of LV volume, cardiac output and E/A wave ratio [153]. Based on these findings in systemic hypertension and LV disease, it seems likely that any therapy targeting cardiac macrophages in PAH might need to be selective, targeting only pathologic macrophage subpopulations. Additionally, the timing of therapeutic interventions relative to disease progression may be relevant.

### Future directions

Studies addressing immune phenotype and functional assessment of the different subsets of macrophages during RV remodelling and failure in patients with PAH are largely unexplored, due in part to the fact that: 1) RV remodelling and failure in PAH-mouse models do not fully reproduce the PAH-human phenotype, and therefore any data related to RV remodelling in knockout mice have to be carefully interpreted; and 2) the study of immune cell phenotyping in PAH-rat models is challenging because the knowledge of rat macrophage cell markers, that would allow the distinction of subsets of RV macrophages, is much less developed than in mice or humans [147]. These limitations underscore the importance of future studies using single-cell sequencing, spatial proteomics and advanced flow cytometry in rat models of PAH and human tissues to better understand the role of different subsets of RV macrophages during the progression of PAH leading to RVF. Controversies in the literature regarding the role of macrophages in PAH reflect (in part) the use of different rodent models of PAH and their fidelity to human disease, suboptimal immune cell phenotyping in rats, and true tissue-specific heterogeneity in the function and pathologic implications of various immune cells. Furthermore, the role of immunometabolism in controlling macrophage function requires rigorous studies in rodents and humans.

Currently, there is a gap in understanding how dynamic changes in metabolism, phenotype and function of RV macrophages, resident or monocyte-derived, influence cardiac cells, including cardiomyocytes, fibroblasts and endothelial cells, leading to the maladaptive remodelling of the RV. A better understanding of the role of resident *vs*. monocyte-derived macrophages is needed, along with the development of selective targeting strategies to better target inflammation in PAH-RVF. It remains to be established whether macrophage metabolism is a major regulator of inflammation, and if so, whether their metabolism can be approached as a therapeutic target to regulate the pathologic function of these cells.

## Conclusions

Macrophage immunometabolism is associated with significant disease-related changes in macrophage phenotype and function. While metabolism is significantly changed in PAH, and can regulate macrophage function, it is not known whether metabolic regulation of macrophages determines the inflammatory phenotype of macrophages in PAH. Research on macrophage metabolism and its relationship to the multiple macrophage phenotypes and origins has been primarily done in macrophages derived from healthy animal models. As such, the applicability to different disease frameworks and environments is uncertain. Further research is necessary to examine the relationship between macrophage polarization and metabolism of macrophages in the lungs and RVs of rodents with experimental PAH, and this needs to be correlated with findings from patient tissues. It appears likely that the macrophages in the RV and lung in PAH are phenotypically distinct, and this tissue heterogeneity may have both mechanistic and therapeutic implications. Reducing the pro-inflammatory phenotype of RV macrophages in PAH through modulation of their metabolism may significantly affect disease outcomes and could reduce RVF, highlighting the importance of further studying immunometabolism in PAH.

## Open Access

This article has been published open access under our Subscribe to Open programme, made possible through the support of our subscribing institutions, learn more here: https://portlandpress.com/pages/open_access_options_and_prices#conditional

## Abbreviations

PAH: pulmonary arterial hypertension
PASMCs: pulmonary arterial smooth muscle cells
PAs: pulmonary arteries
PH: pulmonary hypertension
PVR: pulmonary vascular resistance
RVF: right ventricular failure
